# Histopathologic and immunohistochemical features of capsular tissue around failed Ahmed glaucoma valves

**DOI:** 10.1371/journal.pone.0187506

**Published:** 2017-11-09

**Authors:** Alka Mahale, Fatma Fikri, Khitam Al Hati, Sami Al Shahwan, Ibrahim Al Jadaan, Hind Al Katan, Rajiv Khandekar, Azza Maktabi, Deepak P. Edward

**Affiliations:** 1 King Khaled Eye Specialist Hospital, Riyadh, Kingdom of Saudi Arabia; 2 Wilmer Eye Institute, Johns Hopkins University School of Medicine, Baltimore, MD, United States of America; 3 Department of Ophthalmology and Visual Sciences, University of Illinois at Chicago, Chicago, Illinois, United States of America; Pennsylvania State Hershey College of Medicine, UNITED STATES

## Abstract

Impervious encapsulation around Ahmed glaucoma valve (AGV) results in surgical failure raising intraocular pressure (IOP). Dysregulation of extracellular matrix (ECM) molecules and cellular factors might contribute to increased hydraulic resistance to aqueous drainage. Therefore, we examined these molecules in failed AGV capsular tissue. Immunostaining for ECM molecules (collagen I, collagen III, decorin, lumican, chondroitin sulfate, aggrecan and keratan sulfate) and cellular factors (αSMA and TGFβ) was performed on excised capsules from failed AGVs and control tenon’s tissue. Staining intensity of ECM molecules was assessed using Image J. Cellular factors were assessed based on positive cell counts. Histopathologically two distinct layers were visible in capsules. The inner layer (proximal to the AGV) showed significant decrease in most ECM molecules compared to outer layer. Furthermore, collagen III (p = 0.004), decorin (p = 0.02), lumican (p = 0.01) and chondroitin sulfate (p = 0.02) was significantly less in inner layer compared to tenon’s tissue. Outer layer labelling however was similar to control tenon’s for most ECM molecules. Significantly increased cellular expression of αSMA (p = 0.02) and TGFβ (p = 0.008) was detected within capsular tissue compared to controls. Our results suggest profibrotic activity indicated by increased αSMA and TGFβ expression and decreased expression of proteoglycan (decorin and lumican) and glycosaminoglycans (chondroitin sulfate). Additionally, we observed decreased collagen III which might reflect increased myofibroblast contractility when coupled with increased TGFβ and αSMA expression. Together these events lead to tissue dysfunction potentially resulting in hydraulic resistance that may affect aqueous flow through the capsular wall.

## Introduction

Glaucoma drainage devices are useful in treating refractory glaucoma [1]. Commercially available glaucoma drainage devices (GDDs) are Ahmed (New World Medical, Inc., Rancho Cucamonga, CA, USA), Baerveldt (Advanced Medical Optics, Inc., Santa Ana, CA, USA), Krupin (Eagle Vision, Inc., Memphis, TN, USA) and Molteno implants (Molteno Ophthalmic Ltd., Dunedin, New Zealand). They share a common design consisting of a small caliber silicone tube that is inserted into the eye and drains aqueous humor to an episcleral plate [2]. The episcleral plates of these devices differ in surface area, shape, thickness, the presence or absence of a valve and technique of surgical installation [3]. The overall success rate of these drainage devices appears to be similar in controlling IOP and a major cause of attenuated long-term success is attributed to excessive fibrous reaction of the capsular tissue [4].

The success of drainage devices surgery depends on the formation and maintenance of a permeable capsule around the episcleral plate, through which the aqueous percolates into surrounding tissues by simple diffusion [2, 5, 6]. The capsule around the shunt plate provides the primary resistance to aqueous outflow through the drainage device [7]. As a result, the most important factor in determining the long term intraocular pressure control is the permeability of the capsule surrounding the plate [6, 8, 9]. Progressive capsular fibrosis around the implant and relative impermeability of the shunt capsule in many cases results in clinical failure, necessitating further medical or surgical management.

The tissue related factors that determine the permeability of the capsule have been investigated and although better understood, still remain unclear [10]. Active wound healing after glaucoma shunt surgery results in excessive and persistent ECM deposition particularly collagen compromising capsular permeability [11–13]. Molteno implant capsules have been described to consist two distinct layers. These include a thin external fibroproliferative moderately cellular layer showing small blood vessels and normal appearing collagen as well as an inner (in proximity to the shunt plate) thicker, relatively hypocellular and avascular fibrodegenerative layer with altered collagen [5, 6, 14, 15].

The hydraulic resistance of interstitium influences many aspects of body fluid physiology including fluid drainage from anterior chamber of the eye. Such resistance is attributed to the nature of extracellular matrix that includes the collagens, proteoglycans and glycosaminoglycans (GAGs) [16]. We hypothesized that abnormal expression of extracellular matrix (ECM) proteins and components of tissue fibroblasts may be involved in altered permeability of capsules surrounding the shunt plate and may contribute to the increased hydraulic resistance, and was the basis of this study.

## Materials and methods

### Patients

All patients were seen at King Khaled Eye Specialist Hospital, Riyadh, Saudi Arabia. The medical records of patients who underwent revision of Ahmed glaucoma valve implant (Models S1 and S2, New World Medical, Inc., Rancho Cucamonga, CA) for uncontrolled IOP with maximal tolerated medical therapy were reviewed retrospectively to obtain clinical information where available. Failure was defined as intraocular pressure that was above target levels on maximum medical therapy as determined by the treating physician. Inclusion criteria included patients with poorly controlled IOP above target as determined by the treating physician (range 22–40 mm Hg) where excision of the capsule was deemed, in the opinion of the physician to be beneficial in controlling IOP. Exclusion criteria included neovascular glaucoma or glaucoma where additional factors may influence the tissue response. The revisions were performed at KKESH between year 1995 and 2010. The Ahmed valve revision in all patients involved excision of the varying amounts of capsule surrounding the implant. Archived paraffin embedded tissue blocks from the capsules excised during the revision were retrieved from pathology archives for light microscopy and immunohistochemistry. Control tenon’s tissue obtained during primary insertion of Ahmed valves was processed and embedded in paraffin for the study. Archived tissue blocks were utilized for the study and data was accessed and analysed anonymously.

The study was approved by the Institutional Review Board of King Khaled Eye Specialist Hospital and followed the principles established in the Declaration of Helsinki. The IRB waived the need for consent.

### Histopathology and immunohistochemisty

Formalin-fixed paraffin embedded (FFPE) tissue from excised capsules and control tenon’s tissue were sectioned at 5 μm thickness and mounted on coated glass slides. The sections were stained with haematoxylin and eosin for histological evaluation and thickness of the two layers measured. The thickest area of the inner and outer wall was identified and measured in an area that was well demarcated. The measurement was done using Olympus CELLSENS software measurement tools (Olympus America Inc, Center Valley, PA, USA). Indirect immunohistochemistry was performed using a Dako automated stainer (Autostainer Link 48, Dako, Glostrup, Denmark). Briefly, deparaffinised slides were treated with antigen retrieval solution (Dako, Denmark) in the Pt link module (Dako, Denmark) processed for staining and the reaction was visualized by using Envision Flex Visualization system (Dako, USA). For some samples limited tissue was available during staining, and repeat staining was also not possible in these cases. Hence only those cases were considered where data could be reliably reported and specified accordingly in the results for each antibody. Appropriate positive and negative control tissue were used. Table 1 lists the antibodies used in the study and their role in scarring.

**Table 1:** Antibody markers used in the study.

Antibody	Company (Catalogue #)	Dilution	Label target	Role in scarring
Collagen I	Abcam (ab 34710)	1:500	Extracellular Matrix component (Fibrillar)	Fiber-forming ECM components, provides structural support, remodelled during wound healing, deregulated expression causes tissue dysfunction [17].
Collagen III	Biogenex (ab167-5M)	Ready to use		
Decorin	Abcam (ab115744)	1:100	Extracellular Matrix component (Proteoglycan)	Natural antagonists and regulators of TGFβ activity [18, 19]. Interact with and inhibit collagen fibrillogenesis [20, 21].
Lumican	USBiological (L6025)	1:100		
Aggrecan (chondroitin sulfate proteoglycan 1)	Abcam (ab 3778)	1:50	Extracellular Matrix component (Proteoglycan)	Structural constituents of ECM, among other functions maintain osmotic pressure and proper collagen organization [22], modulates profibrogenic TGFβ signalling [23].
Chondroitin sulfate proteoglycan (CSPG)	Abcam (ab 11570)	1:50	Extracellular Matrix component (Sulphated GAG)	Synthesized by fibroblasts, interfibrillary ECM ground substance, provides lubrication and acts as a spacer between moving collagen fibers, maintains tissue hydration, important for architecture of healing tissue provides structural and regulatory function [24–27].
Keratan sulfate proteoglycan	Iowa (MZ15-S)	1:6	Extracellular Matrix component (Sulphated GAG)	
Alpha smooth muscle Actin (αSMA)	Abcam (ab7817)	1:50	Activated fibroblasts (Myofibroblasts)	Myofibroblasts play key role in normal wound repair and are responsible for wound modulation, wound closure through contraction, secretion of ECM and other pro-fibrotic molecules [28, 29].
TGFβ	Abcam (ab 66043)	1:100	Cellular and secreted	Growth Factor, mediator of fibrosis, controls secretion of ECM molecules [30].

### Quantification of immunohistochemistry with Image J

Digital images were captured using Olympus BX 53 Microscope (Olympus America Inc, Center Valley, PA, USA). The photographs were taken at a standardized exposure time to evaluate and quantify color intensity using ImageJ-color-deconvolution. The deconvolution method has been used to separate the brown DAB chromogen from hematoxylin counterstain on a microscope slide and the measurement of color intensity of specific stains [31, 32]. Briefly, the deconvolved DAB image was subjected to histogram analysis using NIH-ImageJ program (NIH Bethesda, MD, USA). The output of this analysis contains number of pixels at each pixel intensity ranging between 0–255, histogram value in the NIH, Image J histogram list, where 0 was very dark and corresponded to dark/intense staining and 255 was very bright and corresponded to very little staining. The data obtained included the mean, standard deviation, minimum and maximum of values for areas of measurement. The capsule as described previously in the literature [6] had two distinct layers and appeared to have staining that was different in intensity. Therefore a total of at least four areas, two in each of the inner and outer layers of the capsule were sampled during the quantitative assessment in a masked fashion by one observer. Similarly four areas were sampled in control tenon’s tissue and since layer demarcations were absent the values pooled for analysis. αSMA and TGFβ labelling was reported as cell counts.

### Statistical methods

Statistical Package for Social Studies (SPSS-19) IBM Chicago, USA) was used for statistical analysis. Descriptive statistics were used to report demographic characteristics. The intensity of labelling measured was reported as median, 25% quartile and range. The label intensity between cases and control was compared using non parametric analysis and validated by Kruskal Wallis Test. A two sided ‘p’- value of less than 0.05 was considered as statistically significant.

## Results

**Fig 1:**
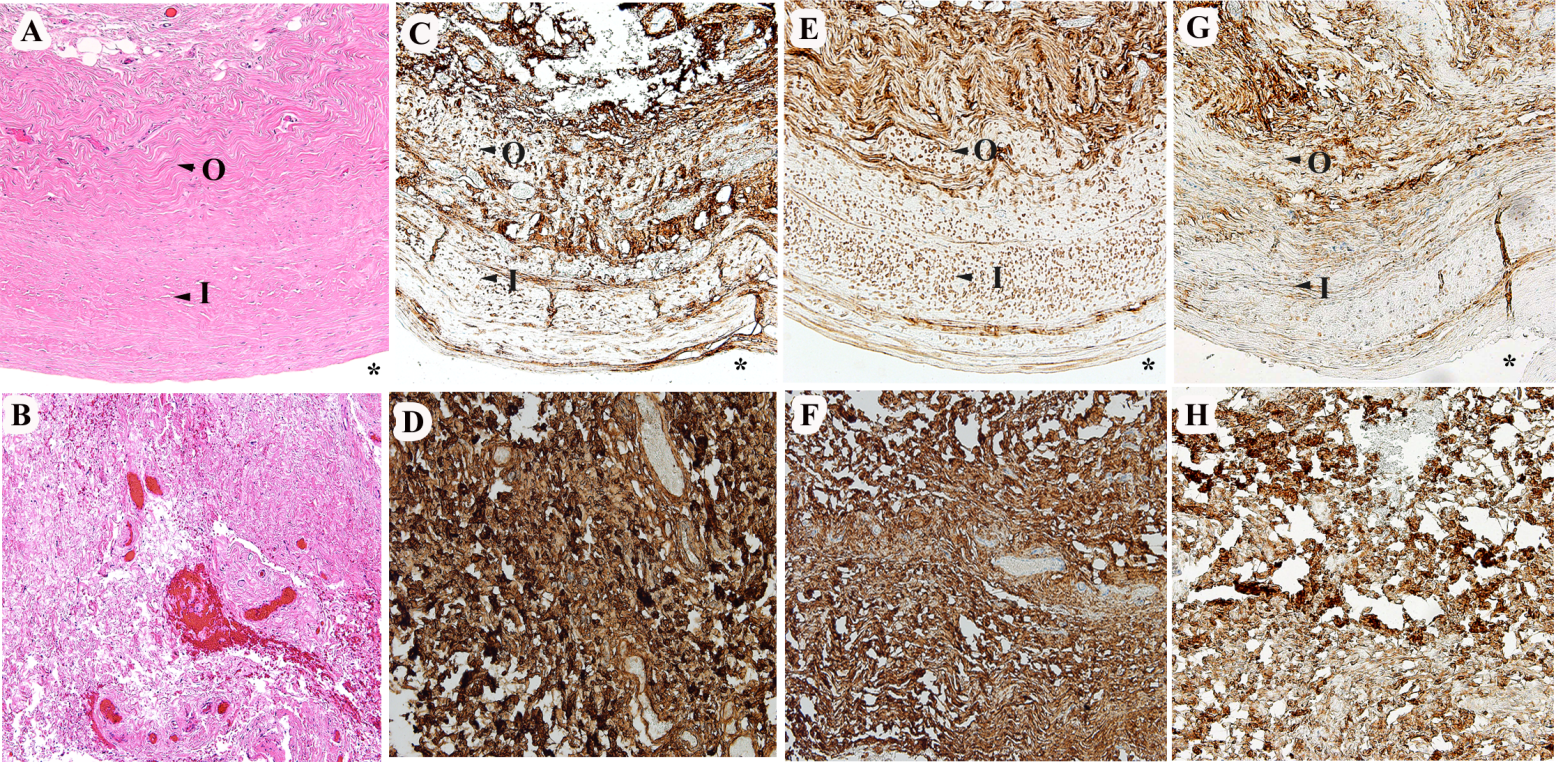
Histology and immunohistochemical staining of select ECM molecules. Excised capsule around Ahmed valve (upper panel) and control tenon’s tissue (bottom panel). Haematoxylin and eosin stained sections (A and B), Collagen III (C and D), Decorin (E and F) and Lumican (G and H). * Indicates bleb cavity around Ahmed valve, O and I mark the inner and outer layers respectively. 100X magnification.

We studied 14 excised AGV capsules and 8 normal tenon’s tissues as controls. The clinical profile of these subjects is summarized in Table 2. The diagnosis included congenital glaucoma (71.4%), secondary glaucoma (14.3%), and primary open angle glaucoma (14.3%). AGV model S2 was inserted in 86%, while 14% received the S1 model. The mean IOP prior to AGV revision was 38.6±11.6 mmHg. The median interval between AGV insertion and valve revision was 13.5 months (Range 3–156; 25% Quartile; 4 months). Excised capsule showed two distinct layers in all examined tissues. This included an outer layer that consisted of loosely arranged collagen bundles, spindle shaped fibroblasts and mature blood vessels that were variable in calibre and an inner layer that was composed of dense compact collagen bundles with several spindle shaped fibroblast and few mature thin walled blood vessels (Fig 1). The mean thickness of the inner and outer layers of the capsule was 444.6 μm and 393.2 μm respectively (Table 2).

**Table 2:** Demographic data and clinical parameters of patients who underwent AGV revision and control (Tenon’s) cases.

		Capsules	Tenon’s
		(n = 14)	(n = 8)
Age (years)	Median (Quartile)	8 (4.75)	24 (18)
	Minimum—Maximum	1–58	1–86
Gender	Male	10 (71%)	5(63%)
	Female	4 (29%)	3(37%)
Diagnosis	Congenital Glaucoma	10 (71.4%)	-
	Secondary Glaucoma	2 (14.3%)	-
	Primary Open Angle Glaucoma	2 (14.3%)	8 (100%)
Type of implant	S1	2 (14%)	-
	S2	12 (86%)	-
Preoperative Glaucoma medications	Median (Quartile)	3 (3)	-
	Minimum—Maximum	2–4	-
Preoperative IOP (mm Hg)	Mean (± SD)	38.6 (±11.6)	-
Interval between primary implant and revision (months)	Median (Quartile)	13.5 (4.0)	-
	Minimum—Maximum	3–156	-
Thickness of capsular layers (μm)	Mean (± SD)		
Inner		444.6 (194.2)	-
Outer		393.2 (163.2)	-
Duration of Follow up after revision (years)	Median (Quartile)	7.8 (4.7)	-
	Minimum—Maximum	0.1–16	-
Final IOP (mm Hg)	Mean (± SD)	22.8 (±9.5)	-

The intensity of immunostaining for all the examined markers in capsules and controls is detailed in Table 3. Both the capsules and controls expressed most of the molecules that were included in this study. To examine whether the ECM markers were differentially expressed in the inner and outer capsular layers, we compared the biomarker density in these layers (Table 3). We observed a statistically significant decrease in labelling in the inner layer of the capsule for ECM molecules collagen I (p = 0.0002) and collagen III (p = 0.0002), decorin (p = 0.0003), lumican (p = 0.01), chondroitin sulfate (p = 0.0002), aggrecan (p = 0.01) and keratan sulfate (p = 0.01) (Table 3). Having found these differences, we compared ECM staining separately for each inner and outer layer between the capsules and controls. Notably, significant differences were observed in the inner layer compared to control tenon’s tissue. This included a significantly decreased expression of collagen III (p = 0.004), decorin (p = 0.02), lumican (p = 0.01) and chondroitin sulfate (p = 0.02) in the inner layer of the capsule (Table 3 and Fig 1). Expression trend of ECM in the outer layer was similar to the controls and differences were not statistically significant except with collagen III (p = 0.04) and keratan sulfate (p = 0.03) where lower expression was seen in the outer layer of the capsule (S1 Table).

**Table 3:** Summary of immunolabels and differences in biomarkers in inner and outer layers of the excised capsules and in control tenon’s.

		Median (Minimum—Maximum)	25% Quartile	KW test (p value)^a^	KW test (p value)^b^
Collagen I	Capsule outer layer	163.8 (106–184)	146.2	0.0002^c^	
n = 14	Capsule inner layer	177.9 (151–201)	166.3		0.9
	Tenon	174.3 (149.2–205.6)	154		
Collagen III	Capsule outer layer	145.6 (118–173)	129.6	0.0002^c^	
n = 14	Capsule inner layer	184 (138–216)	165.1		0.004 ^c^
	Tenon	95.2 (60.7–222.3)	66.7		
Decorin	Capsule outer layer	132.1 (81–202)	92.7	0.0003^c^	
n = 13	Capsule inner layer	190.9 (124–225)	152.6		0.02^c^
	Tenon	138.1 (69.7–205.4)	102.7		
Lumican	Capsule outer layer	146.4 (107–172)	119.3	0.01^c^	
n = 12	Capsule inner layer	190.4 (154–214)	166.1		0.01^c^
	Tenon	150.6 (105–194)	138.3		
Chondroitin sulfate	Capsule outer layer	157.3 (134–188)	143.5	0.0002^c^	
n = 14	Capsule inner layer	215.4 (169–235)	203.3		0.02^c^
	Tenon	200.7 (106.1–230.2)	179		
Aggrecan	Capsule outer layer	211.1 (182–240)	197.5	0.01^c^	
n = 12	Capsule inner layer	214.4 (183–240)	203.3		1.0
	Tenon	218.1 (188.0–223.1)	204		
Keratan sulfate	Capsule outer layer	196.3 (184–203)	189.4	0.01^c^	
n = 12	Capsule inner layer	198.9 (191–210)	196.4		0.08
	Tenon	191.8 (150.2–209.4)	187		
αSMA	Capsule	17 (3–50)	11.5		0.02 ^d^
n = 13	Tenon	5.3 (2–23)	2		
TGFβ	Capsule	210.4 (94.5–288)	59.0		0.008 ^d^
n = 10	Tenon	64.3 (12.3–245)	37.5		

All markers except αSMA and TGFβ were as graded by the Image J software in the inner and outer layers of the capsule and control tenon’s tissue. Note that a lesser value indicates greater intensity of label for these molecules. n = 10–14 cases for excised capsules as specified above for each antibody, n = 8 for control tenon’s tissue except for lumican where n = 7. Kruskal Wallis test, two sided *p* value was performed to validate differences in label intensity between inner and outer capsular layers (a), and between inner capsular layer and control tenon’s (b) with significantly lower expression in the inner capsular layer (c), αSMA and TGFβ are reported as manual cell counts with significantly higher positivity in the excised capsules (d).

**Fig 2:**
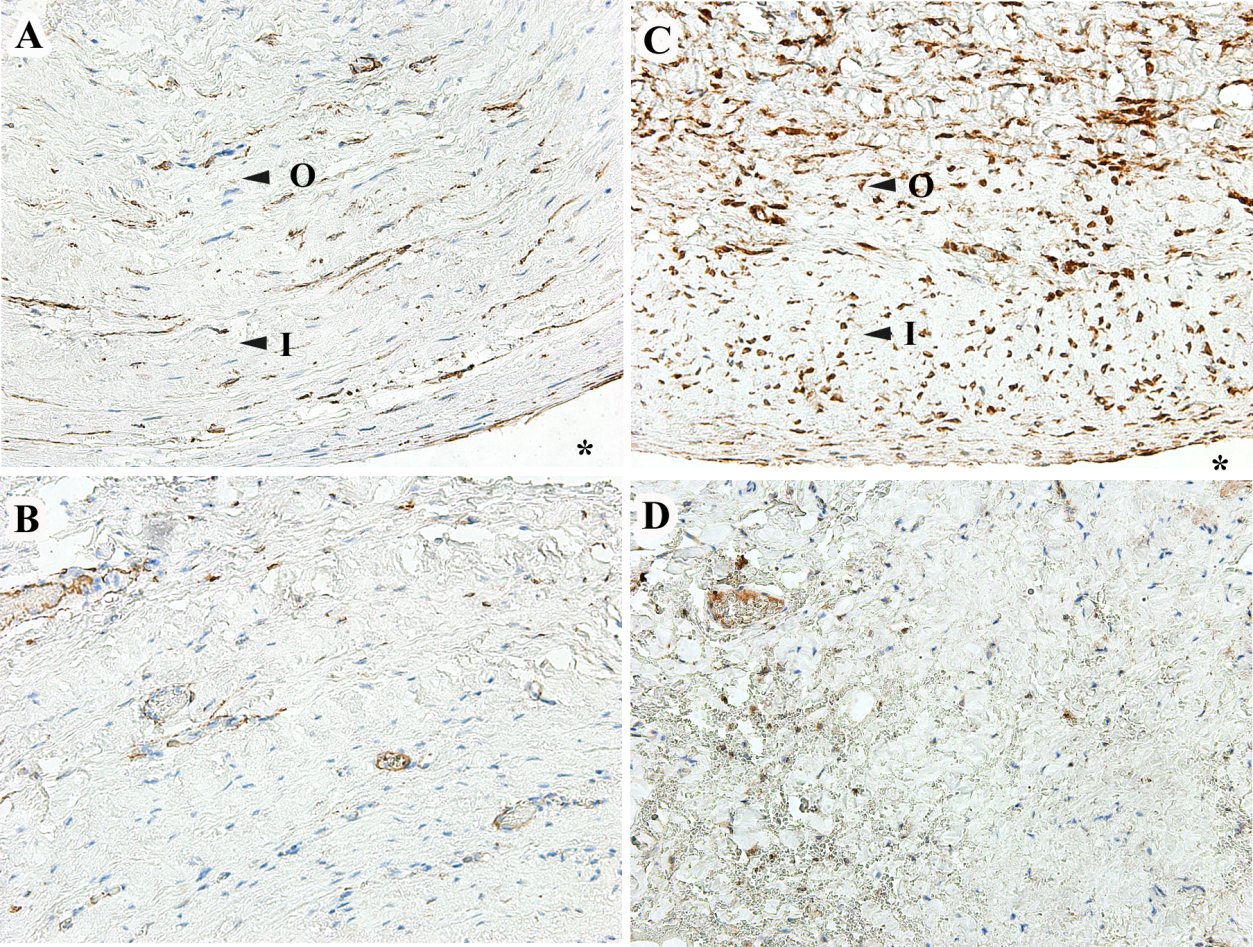
Immunohistochemical staining for αSMA and TGFβ. Excised capsule around Ahmed valve (upper panel) and control tenon’s tissue (bottom panel) for αSMA (A and B), TGFβ (C and D). *Indicates bleb cavity around Ahmed valve, O and I mark the inner and outer layers respectively. 200X magnification.

We also observed increased cellular expression of αSMA (p = 0.02) and TGFβ (p = 0.008) in the fibroblasts within the capsules compared to the controls (Table 3). The distribution of the marker positive cells was not layer specific and not limited to either inner or outer layers of the capsule (Fig 2).

## Discussion

Fibrosis of the capsule surrounding a glaucoma implant remains the main cause of suboptimal pressure control and failure of drainage procedures [33]. In this study, we examined fibrous capsules surrounding failed Ahmed implant for expression of a number of extracellular matrix proteins and cellular markers. We demonstrated a consistent trend of altered expression of molecules that suggest profibrotic activity as indicated by increased expression αSMA and TGFβ, together with decreased expression of ECM proteoglycan/GAGs such as decorin, lumican and chondroitin sulfate as well as fibrillar collagen III. It was surprising to note altered expression of several molecules in the excised capsule several months/years following shunt implantation.

Histologic findings in the capsular tissue were similar to previous reports which described an outer more vascular layer with loosely arranged matrix and an inner relatively avascular layer with dense connective tissue [6, 34]. The outer layer however was similar to control tenon’s in terms of histopathological characteristics as well as expression trend for most of ECM molecules used in this study. Tenon’s capsule is a vascular connective tissue that drapes the shunt plate, absorbs and drains aqueous in the initial period after surgery [6, 35]. It is believed that tenon’s tissue fibroblasts contribute largely to the encapsulation and several studies have utilized tenon’s tissue fibroblasts to understand bleb failure after glaucoma surgery [36–40]. Therefore as a baseline tenon’s control helps to provide the evidence to speculate several potentially important interactions.

Increased expression of αSMA, an indicator of activated myofibroblasts which are key effector cells of fibrosis, was seen in our samples as reported [11, 12]. The presence of this smooth muscle protein is linked to contractile nature of myofibroblasts, which when persistent can cause distortion of tissue architecture, thus promoting disease pathogenesis [41]. We also demonstrated increased expression of TGFβ protein, a known inducer of myofibroblast transformation [42], [43], and provide evidence for the presence of an intrinsic trigger in the capsular tissue. It has been suggested that growth factors including TGFβ present in aqueous humor might trigger the fibrotic response [44–46]. However, our data is consistent with a recent study in which our group showed an increase in transcripts for matrix molecules as downstream targets of activated TGFβ pathway [47]. Saika et al. have also reported increased TGFβ protein expression in tissue from a trabeculectomy filtering bleb [48].

Accompanying increased expression of TGFβ in our samples was the decrease in ECM molecule decorin. Proteoglycan decorin is a naturally occurring TGFβ antagonist [18] that prevents fibrosis and improves surgical outcome of glaucoma filtration surgery in rabbits [49]. Additionally, we also observed a significantly decreased labelling with lumican, chondroitin sulfate and collagen III. Like decorin, lumican is also an endogenous inhibitor of TGFβ activity [19] which binds TGFβ receptor 1 (ALK5) and downregulates TGFβ signalling [50]. Although the mechanisms have not yet been defined, lumican has been implicated in regulating aqueous humor outflow in the trabecular meshwork [51, 52]. Furthermore, we and others have shown increased levels of MMPs in capsular tissue [47, 53]. It is known that degradation and cleavage of decorin and lumican is induced by matrix metalloproteinases (MMPs), a family of proteinases that can cleave extracellular matrix molecules [54, 55]. Degradation of these proteoglycans by MMPs may also explain the decreased decorin and lumican seen in our samples. Thus, besides increased expression, downregulation of these endogenous inhibitors might be another mechanism for activation of the profibrotic TGFβ signal in the capsular tissue.

Furthermore, decrease in decorin and lumican might influence the assembly of collagen in the inner capsular layer. In normal cornea lumican maintains orderly collagen fibril arrangement that is vital for corneal transparency [56–58]. In contrast down regulation of both decorin and lumican result in thicker and mal-oriented collagen fibres as well as decreased inter fibrillar distance in the corneas of knockout models [59, 60]. Morphology of opacified corneal stroma in knockout mice bears some resemblance to that seen in the inner layer of the excised capsules where compact, thick irregularly arranged collagen fibres are observed. Due to their role in collagen fibrillogenesis and wound healing, decreased expression of chondroitin sulfate glycosaminoglycans as seen in our capsules might also structurally alter the collagen scaffold [61, 62]. We also found significantly decreased expression of collagen III in the inner layer of our capsular samples. Fibrillar collagens form a major component of the ECM and diminished collagen III expression in haploinsufficient (Col III+/–) mice has been reported to accelerate cutaneous wound closure by promoting myofibroblast differentiation and increased scar formation [63].

We recognize that our study has limitations and further investigations will be needed in this regard. Capsules from functional AGV devices would probably be ideal second comparative controls for this study. However, functional capsules are not commonly excised and were not available for this study. Also, capsules examined in our study were mostly late excisions and expression changes in early stages of shunt failure could not be determined. It has been suggested that thickness and permeability of capsule around the implant is regulated by inflammatory/proliferative and apoptotic processes occurring during wound remodelling of the capsular tissue as a result of exposure to glaucomatous aqueous humor [64, 6]. However, in capsular tissue excised several years after implantation, we did not observe an inflammatory response. Furthermore, although clinical observation suggests that tenon’s capsule thins with age and measured by optical coherence tomography (OCT) is variable in thickness [65]. Whether this change in physical characteristics of the capsule affects molecular changes is unclear. Nevertheless, tenon's fibroblasts derived from young versus old human eyes do show growth differences in vitro; however, wound closure/migration and collagen synthesis rates were reported to be similar [66]. Increasing the sample size while utilizing age matched capsular and tenon’s tissue in future studies would likely give better statistical power to allow for correlation of expression changes with clinical parameters such as age, type of glaucoma, revision time and other variables. TGFβ is a pleiotropic molecule with complex roles, its relation to other pathways affecting glaucoma surgery such as inflammation and angiogenesis also requires further investigations [67, 68].

ECM has structural functions and increased expression of ECM molecules could be expected to explain the hydraulic resistance. Paradoxically, we found decreased expression of proteoglycans (decorin and lumican), GAGs (chondroitin sulfate) and collagen III. Together with increased TGFβ and αSMA this decrease of ECM molecules might indicate interplay of molecules potentially sustaining a profibrotic environment leading to myofibroblast contractility and tissue dysfunction. Such a role for the ECM as regulators of cell signalling is receiving increasing support [69, 70] and research in this area has demonstrated the importance of re-establishing a functional ECM in chronic wounds. Although our study points out an important role for ECM molecules, much remains to be understood in the context of wound healing in glaucoma filtration surgery to decrease outflow resistance and improve long-term IOP control.

## Supporting information

S1 Table Difference of biomarker density between outer capsular layer and in control tenon’s. (DOCX)

S2 Table Minimal data set. (XLSX)
