# Supplementary material for: Histopathologic and immunohistochemical features of capsular tissue around failed Ahmed glaucoma valves
Source: PLoS One. 2017 Nov 9;12(11):e0187506. doi: 10.1371/journal.pone.0187506 (PMC5679546; doi:10.1371/journal.pone.0187506)
Supplement: S1 Table — (DOCX) [file pone.0187506.s001.docx]

**S1 Table. Difference of biomarker density between outer capsular layer and in control tenon’s**

|  | Outer layer of excised capsule (n=14) | | Control tenon’s tissue (n=8) | |  | Comments |
| --- | --- | --- | --- | --- | --- | --- |
|  | Median (Minimum - Maximum) | 25% Quartile | Median (Minimum - Maximum) | 25% Quartile | KW* test (p value) |  |
| Collagen III | 145.6 (118 - 173) | 129.6 | 95.2 (60.7 - 222.3) | 66.7 | 0.04 | Lower in outer layer of excised capsule |
| Keratan sulfate | 196.3 (184 – 203) | 189.4 | 191.8 (150.2 - 209.4) | 187 | 0.03 | Lower in outer layer of excised capsule |
| Collagen I | 157.3 (134 – 188) | 143.5 | 174.3 (149.2 - 205.6) | 154 | 0.64 |  |
| Decorin | 204.2 (192 – 218) | 197.0 | 138.1 (69.7 -205.4) | 102.7 | 0.35 |  |
| Lumican | 146.4 (107 – 172) | 119.3 | 150.6 (105 – 194) | 138.3 | >0.9 |  |
| Chondroitin sulfate | 132.1 (81 – 202) | 92.7 | 200.7 (106.1 - 230.2) | 179 | 0.27 |  |
| Aggrecan | 211.1 (182 – 240) | 197.5 | 218.1 (188.0 - 223.1) | 204 | 0.92 |  |

* Kruskal Wallis Test, two sided *p* value.
